# Metastatic ameloblastoma responding to combination chemotherapy: case report and review of the literature

**DOI:** 10.1186/1752-1947-5-491

**Published:** 2011-10-03

**Authors:** Mounia Amzerin, Zouhour Fadoukhair, Rhizlane Belbaraka, Meryem Iraqui, Saber Boutayeb, Hind M'rabti, Tayeb Kebdani, Khaled Hassouni, Najib Benjaafar, Brahim K El Gueddari, Hassan Errihani

**Affiliations:** 1 Department of Medical Oncology, National Institute of Oncology, Rabat, Morocco; 2 Department of Radiotherapy, National Institute of Oncology, Rabat, Morocco

## Abstract

**Background:**

Ameloblastoma is a rare benign odontogenic tumor with locally aggressive behavior and a high recurrence rate. When metastases occur, which are uncommon, lungs constitute the most frequent site involved. Malignant ameloblastomas are different from ameloblastic carcinomas. Malignant ameloblastomas are tumors considered metastatic despite the appearance of well-differentiated or benign histology, while ameloblastic carcinomas are histologically malignant in both primary and metastatic sites.

**Case presentation:**

A 24-year-old Moroccan man presented a malignant ameloblastoma of the mandible. The tumor was entirely resected. Five years later, a local recurrence occurred. Our patient was treated by exclusive radiotherapy with persistence of a residual disease. After two years he developed multiple lung metastases. Our patient received a combination chemotherapy using doxorubicin and cisplatin.

**Conclusion:**

Less than 50 cases of ameloblastoma with metastases have been reported. There is still no standard treatment for metastatic ameloblastoma. Only through continuous reporting of such cases will clinicians be able to draw an optimal strategy for management of this pathology.

## Introduction

Ameloblastoma, from the English word "amel" which means enamel and the Greek word "blastos" which means germ [1], is a rare entity of benign odontogenic tumor. It arises from the epithelium of the dental lamina and it is known by its local aggressive behavior and the high recurrence rate [2].

Ameloblastoma was first described in 1827 by Cusack [3]. In 1885, Malassez introduced the name "adamantinoma", which is now used to describe a rare form of bone cancer described by Fisher in 1913 [4]. It was renamed to its current denomination by Churchill in 1930 [5].

In the recent WHO classification, a distinction was made between ameloblastoma, malignant ameloblastoma and ameloblastic carcinoma [2]. Malignant ameloblastoma differs from ameloblastoma due to the presence of metastases. They both have the same benign histology [6]. Ameloblastic carcinoma has malignant cytologic features regardless of the presence of metastases. In ameloblastoma, metastases are uncommon. When they occur, lungs are involved in over 80% of cases [7].

Localized disease is treated by radical surgery. However, in metastatic settings, chemotherapy remains the only choice of treatment. Unfortunately, results are unpredictable. We report below a case of an ameloblastoma with metastatic evolution five years after initial surgical treatment.

## Case report

**Figure 1:**
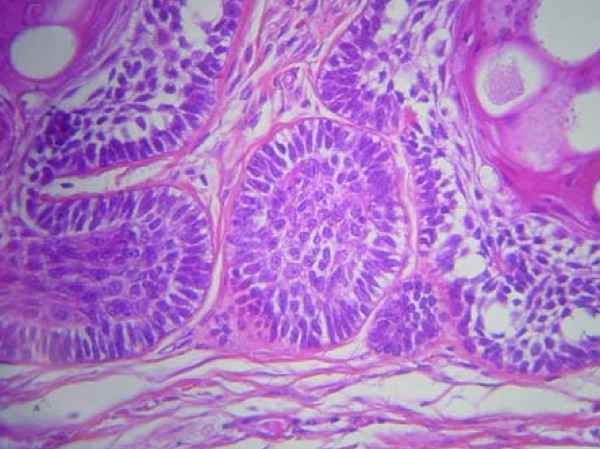
**Histopathologic features of ameloblastoma**.

**Figure 2:**
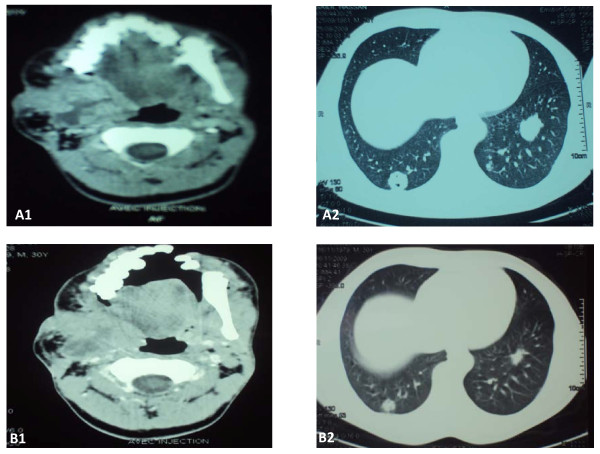
**Presenting symptoms two years after radiothereapy**. **(A) **Before chemotherapy 1 - right submandibular mass with endocranial extension, sphenoidal lysis and infiltration of the hemitongue; 2 - multiple lung metastases. **(B) **After two cycles of chemotherapy. 1 - local stabilization; 2 - partial response.

A 24-year-old Moroccan man presented in 2000 with a mass of the right mandible. A panoramic radiograph revealed a multilocular radiolucency, requiring a biopsy. A histopathological examination of the specimen showed a well-differentiated neoplastic proliferation. This appeared as strands of peripheral columnar cells in palisading orientation. The fibroblastic tumor-associated stroma was dense with collagen fibers and highly infiltrated by inflammatory mononuclear cells. No histological signs of malignancy were observed (Figure 1). The diagnosis of ameloblastoma was confirmed. Treatment consisted of hemimandibulectomy. Surgical margins were free of tumor. Five years later, the lesion recurred as a mass of his right jaw. The recurrence was confirmed by a second biopsy. At the same time, a chest tomography revealed three metastatic nodules of lungs. Our patient received exclusive radiotherapy for the jaw mass, at the dose of 60 Gy. No treatment was delivered for the lung metastases. The disease was controlled for two years, until our patient presented again with a right submandibular mass and multiple bilateral lung metastases (Figure 2A). Our patient received combination chemotherapy using doxorubicin 50 mg/m^2 ^and cisplatin 100 mg/m^2^. The assessment of response to chemotherapy was made after two cycles. The pain disappeared, and tomography showed, according to RECIST criteria, local stabilization and partial response of the lung lesions (30%) (Figure 2B). The response was maintained after six cycles of chemotherapy.

## Discussion

Odontogenic neoplasms are rare tumors of the oral cavity. Among these, ameloblastoma is the second most common entity after odontoma [2]. Ameloblastoma and metastatic ameloblastoma are different. According to the recent WHO classification, ameloblastoma is a localized benign disease whereas malignant ameloblastoma is considered metastatic despite the appearance of well differentiated or benign histology. Ameloblastic carcinoma shows malignant features in both primary and metastatic sites [6].

The average age at diagnosis of patients with ameloblastoma is 34 years with a range of five to 74 years [8]. Men and women are equally affected. The most frequent primary site is the angle of the mandible.

There are several histological subtypes, including plexiform, follicular, acanthomatous, basaloid, granular cell, cystic and desmoplastic forms. So far, the natural course of the disease can not be predicted. The follicular entity is the most common [2]. Basic symptoms are a swelling mass, pain and fistula in the palate [9].

Ameloblastoma is described as a slowly growing, locally invasive benign tumor with a high propensity for local recurrence. There is a 50% to 72% incidence of local recurrence after initial therapy [7]. Radical surgery remains the mainstay of therapy. This is often difficult because of the anatomical complexity of the mandibular region. In case of incomplete resection, radiotherapy can be considered as an adjuvant measure.

Metastases are uncommon, which is why metastatic ameloblastoma is considered benign (in addition to the benign features on histology). They generally occur after an interval ranging from 10 to 12 years [8]. Lungs are involved in 75% to 80% of cases. Other sites may also be involved, such as regional lymph nodes, pleura, vertebra, skull, diaphragm, liver and parotid glands [6]. Many factors have been associated with the tendency to develop metastases, including extent of initial disease, multiple surgeries or radiation therapy [6-8]. Several theories have been suggested to explain metastatic spread, relating to lymphatic or hematogenous causes, aspiration or heterotopia [10].

The treatment for metastatic ameloblastoma remains delusive. When metastases are removable, surgery is the treatment of choice. Results of radiotherapy and/or chemotherapy are unpredictable and the data are poor. Less than 50 cases were reported and conclusions are disparate (Table 1). Gall tested cyclophosphamide and methotrexate 5-fluorouracil therapy in a patient with lung metastases that occurred nine years after initial therapy. He noticed that the functional outcome was good although no objective response was seen [11]. Ramadas obtained partial response after 13 cycles of combination chemotherapy-associating cisplatin and cyclophosphamide administrated for lung metastases [12]. Some other therapies have also shown activity, including vinblastine, bleomycin, paclitaxel and carboplatin [9-13].

**Table 1:** Published data concerning chemotherapy regimens used in metastatic ameloblastoma

Publication details	Regimen	Results	Reference
**CR**	Cycolphosphamide	Good functional	[11]
-M	Methotrexate	outcome	
-Lung metastases	5 Fluorouracil	OR: 0%	

**CR**	Vinblastine	Partial response: 50%	[13]
-M	Cisplatin		
-Lung metastases	Bleomycin		

**CR**	Adriamycin	Partial response	[12]
-W, 17 years old	Cisplatin		
-Lung metastases	Cyclophosphamide		

**CR**	1^st ^line: 5FU-Cisplatin	Progression disease after two cycles; repetitive partial response.	[9]
-W, 28 years old	2^nd ^line: Paclitaxel-		
-Lung metastases	Carboplatin		

**CR**	Cyclophosphamide	OR: 0%	[17]
-M, 46 years old			
-Lung metastases + mediastinal adenopathies			

**CR**	No chemotherapy	Stable disease during 18 months	[6]
-M, 55 years old			
-Lung metastases			

**CR**	No chemotherapy; surgery for removable metastases.	Survival: 54 years	[15]
-W, 39 years old			
-Lung metastases			

**Review**	Doxorubicin ± 5FU.	OR: 0%.	[14]
Outcome of chemotherapy in metastatic ameloblastoma.	Methotrexate ± cyclophosphamide		
	Blemomycin		
	5FU-Cisplatin		
	Vincristine		
	Prednisolone		

CR: case report; M: man; W: woman; OR: objective response.

A review of the literature made by Lanham concluded that chemotherapy failed to show any antitumoral activity, including doxorubicin, methotrexate, prednisolone, bleomycin, 5-fluorouracil and dacarbazin [14]. Moreover, the literature reports some patients with metastases showing long survival without receiving chemotherapy [6,15].

Our case illustrates the natural course of the disease. In spite of radical surgery, lesions recurred. The metastases were asymptomatic. They were discovered during a classical work-up. Our finding, concerning response to doxorubicin-cisplatin, is adding to the published evidence that platinum chemotherapy is active in metastatic ameloblastoma. Moreover, even though no objective response is seen, data show that chemotherapy improves clinical symptoms [6].

There are too few cases of metastatic ameloblastoma to consider randomized trials. Platinum-based regimens could be proposed as a first line treatment. Another pathway to explore is epidermal growth factor receptor (EGFR)-targeting. Ameloblastoma is a tumor originating from EGFR-expressing odontogenic epithelium, with expression levels ranging from 0% to 100% in some studies [16].

## Conclusion

Despite the slowly growing nature of ameloblastoma, endocranial extension and/or occurrence of metastases cause pain and affect survival.

In metastatic ameloblastoma, results are unpredictable. Surgery, when feasible, remains the mainstay of therapy. There is no sufficient data to support or reject the use of chemotherapy. The expression of EGFR by odontogenic tumors could be an interesting approach to explore.

## Consent

Written informed consent was obtained from our patient for publication of this case report and any accompanying images.

## Competing interests

The authors declare that they have no competing interests.

## Authors' contributions

MA performed literature review, the composition of this case report and manuscript writing.

ZF, RB and MI were involved in the conception and design, collection and assembly of the data.
